# Investigation of Genome Biology by Synthetic Genome Engineering

**DOI:** 10.3390/bioengineering10020271

**Published:** 2023-02-20

**Authors:** Hui Zhang, Yao Xiong, Wenhai Xiao, Yi Wu

**Affiliations:** 1 Frontiers Science Center for Synthetic Biology (Ministry of Education), Tianjin University, Tianjin 300072, China; 2 Key Laboratory of Systems Bioengineering (Ministry of Education), School of Chemical Engineering and Technology, Tianjin University, Tianjin 300072, China

**Keywords:** synthetic genome, genome biology, genome engineering

## Abstract

Synthetic genomes were designed based on an understanding of natural genomic information, offering an opportunity to engineer and investigate biological systems on a genome-wide scale. Currently, the designer version of the *M. mycoides* genome and the *E. coli* genome, as well as most of the *S. cerevisiae* genome, have been synthesized, and through the cycles of design–build–test and the following engineering of synthetic genomes, many fundamental questions of genome biology have been investigated. In this review, we summarize the use of synthetic genome engineering to explore the structure and function of genomes, and highlight the unique values of synthetic genomics.

## 1. Introduction

For a long time, knowledge of genome biology was derived from genetic and biochemical analysis of natural cells. With the development of DNA synthesis and assembly technology, it is possible to design and synthesize genomes from scratch. This bottom-up strategy allows us to rewrite DNA sequences on a genome-wide scale, and explore the principles of biology from the perspective of the creator.

**Figure 1:**
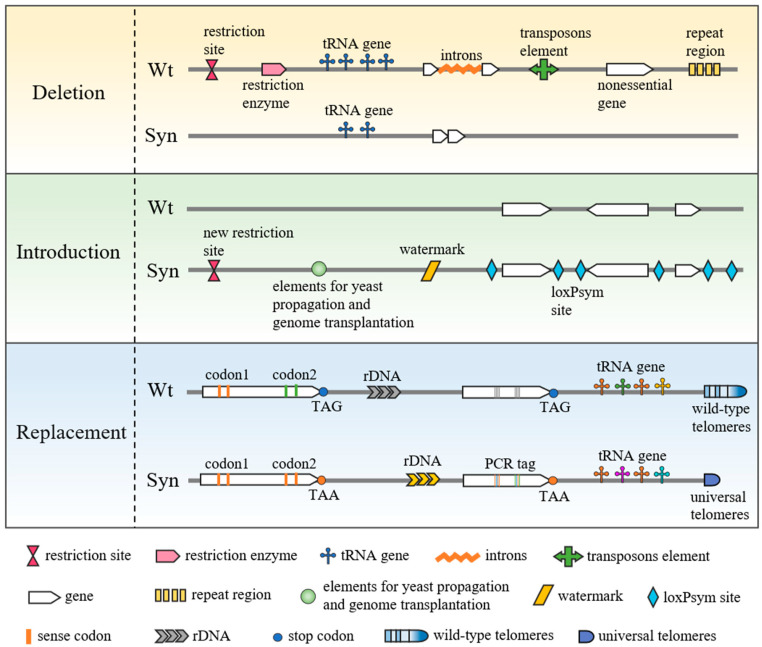
General design principles of synthetic genomes. Nonessential sequence is deleted. Elements are introduced which are required to assemble or research. Replacements are designed according to the purpose of the study.

The field of synthetic genomics has been developed from the small genome scale to the large genome scale, and simple modification to systemic rewriting. Viruses were synthesized first [1,2,3,4,5]. In T7 bacteriophage design, 28.8% of the sequence of the wild-type genome was replaced with an engineered sequence [4]. This means that natural genomes can be designed and rewritten. After that, genomes of organisms which can self-replicate continuously, including *M. mycoides* [6,7], *E. coli* [8,9] and a large proportion of *S. cerevisiae* [10,11,12,13,14,15,16,17,18], were synthesized. Although the purposes of different synthetic genomes are varied, there are some general design principles shown in Figure 1. (1) Deletion: delete endonuclease sites and endonucleases, transposable elements, and some repetitive regions. The purpose of deletion falls into two categories: one is to delete endonuclease sites and endonucleases to facilitate molecular operation; the other is to remove transposable elements and repetitive regions, including subtelomeric repeated sequences and repeated nonessential regions to improve assembly fidelity and genome stability. Additionally, many introns were deleted from the synthetic yeast genome. Considering that only 5% of yeast genes have introns, the synthetic yeast genome provides an opportunity to investigate the significance of intron function. (2) Introducing: insert artificial elements such as restriction sites, watermarks, elements of replication and transplantation, and designer recombination sites. Restriction sites and watermarks facilitate the assembly and identification of synthetic fragments. Since some genomes need to be assembled in yeast and then transplanted to host cells, it is necessary to insert selection and replication elements of yeast and the shuttled elements for transplantation. Other artificial elements may also be inserted for different research purposes. For example, for study of genomic structural variation, a large number of recombination sites, loxPsym, were added to the Sc2.0 design. (3) Replacement: replace the elements such as tRNA, rDNA, synonymous codons, etc. The majority of synthetic genome designs involve the replacement of synonymous codons. It is cumbersome to rewrite codons at the genome-wide scale through traditional engineering methods, but chemical synthesis and assembly of redesigned genomes provide a new method for the study of codon usage.

With rapid development of DNA synthesis, assembly, and transplantation technology [19,20,21], an increasing number of synthetic chromosomes or large genomic segments have been designed and engineered to investigate genome biology. Synthetic genomes can be artificially designed and chemically synthesized without some of the constraints of the native genomes. These synthetic cells provide a unique perspective on many aspects of genome biology [8,22]. While some synthetic genomes cannot support cell survival without the natural genome [9,23,24], the process of debugging can still uncover the function and structure of genomes. In this article, we reviewed the design and engineering of the representative prokaryotic and eukaryotic synthetic genomes and used them as platforms to investigate genome biology.

## 2. Genome Research Using Minimal Synthetic Bacterial Genomes

**Figure 2:**
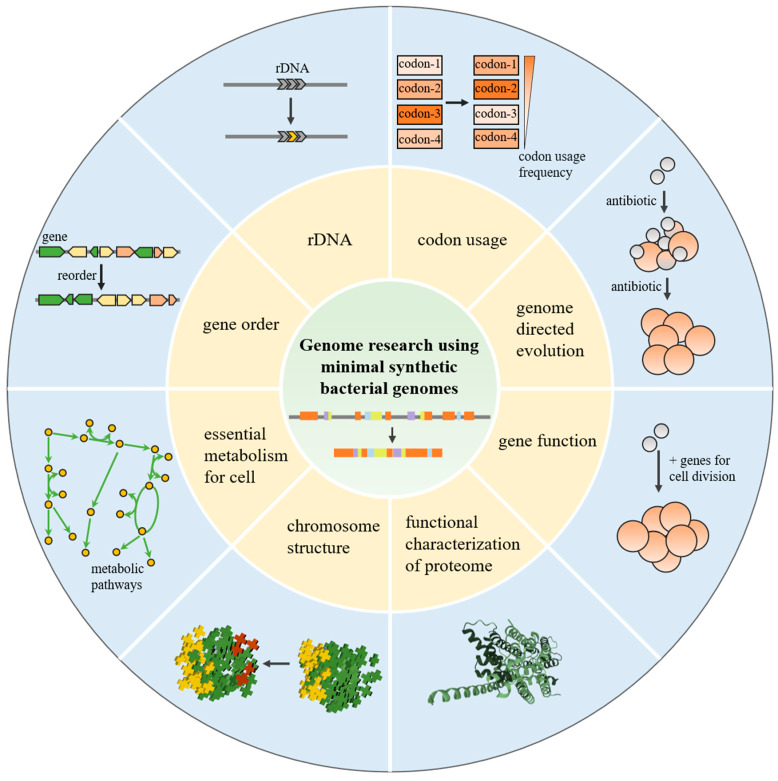
Genome research using minimal synthetic bacterial genomes. Part of the genome was rewritten to explore genome plasticity. Strains can evolve rapidly in an antibiotic environment. Genes for cell division were discovered. A comprehensive cell model was depicted.

In 2010, a new *M. mycoides* cell, JCVI-syn1.0, was created by assembling a synthetic *M. mycoides* chromosome in yeast and transplanting into a *M. capricolum* recipient cell [6,7]. This implies that it is feasible to synthesize and engineer the genome of *M. mycoides*. Considering that *Mycoplasmas* is the simplest cell capable of autonomous growth, JCVI-syn1.0 is an ideal platform to study genome minimization (Figure 2). The most straightforward method to reduce genomes is to design a theoretically minimized genome using existing gene annotations and databases. A theoretical minimal genome was designed according to classification of gene essentiality and then chemically synthesized as eight segments [25,26]. Nevertheless, this design does not produce a viable cell. We still do not fully comprehend the function of genes and their interaction. A strategy of mutagenesis, using Tn5 inserted into JCVI-syn1.0, was proposed to explore gene essentiality. After 4 cycles of mutagenesis, the genes were divided into 5 classification groups: 240 essential genes that were not mutated at all, 48 quasi-essential genes whose deletion might lead to a severe growth defect, 126 genes whose deletion might lead to growth impairments, 55 quasi-nonessential genes whose deletion might lead to minimal growth defects, and 432 nonessential genes for which mutagenesis was frequent. Researchers designed a new genome (RGD1.0) according to Tn5 mutagenesis, including essential and quasi-essential genes. Like JCVI-syn1.0, this genome was also divided into eight fragments for synthesis. When several of these segments were transformed into the genome, strains containing one synthetic segment were viable. However, strains containing the total synthesized genome were not viable. Researchers mixed the eight RGD1.0 segments with the eight JCVI-syn1.0 segments and assembled combinatorial genomes to discover synthetic lethal pairs. After two rounds of combinatorial assembly of genomes and a new round of Tn5 mutagenesis, JCVI-syn3.0 was produced, with a single circular chromosome of only 543 kbp [22]. This is smaller than that of any autonomously replicating cell found in nature. Compared with 901 genes in JCVI-syn1.0, JCVI-syn3.0 contains only 473 genes, including 438 protein- and 35 RNA-coding genes. A total of 195 genes are involved in the process of the expression of genetic information as proteins, such as transcription, translation, and related metabolism regulation; 34 genes are involved in the process of preservation of genome information, such as DNA replication and inheritance and cell division; 84 genes are involved in the process of cell membrane structure and function; and 81 genes are involved in the process of cytosolic metabolism. Of the remaining 79 genes, 65 have unknown functions and 24 are classified as generic. Although it contains only 473 genes, JCVI-syn3.0 has previously identified some genes related to antibiotic tolerance and persistence. Major functions of these genes are involved in global regulation, ATP synthesis, translation, and other important life processes. JCVI-syn3.0 rapidly develops antibiotic tolerance, persistence, and resistance to antibiotics by mutations of genes. This could provide insight into how the first antibiotic genes were generated [27].

The design of JCVI-syn3.0 also attempts to change gene sequence and order. To explore whether the engineered 16S rDNA (rrs) could support cell survival, seven single- nucleotide modifications were introduced into a single copy of the JCVI-syn3.0 rrs gene, and helix h39 (35 nucleotides) was replaced by a counterpart from *E. coli* and other variants. Cells containing helix h39 from *E. coli* were viable without noticeably affecting the growth rate, while the rrs gene of other variants proved nonviable. In the *M. mycoides* genome, TGA encodes tryptophan rather than using it as a stop codon, as in most organisms. To understand the codon usage principles in the *M. mycoides* genome, a 5 kbp region containing 3 essential genes was recoded with the *E. coli* codon adaptation index (CAI) and standard codon usage. Cells containing the recoded region grew normally. To explore the importance of gene order in genome function, genes at segment 2 were reordered. Based on their functions, genes were divided into seven groups: DNA repair, transcription, translation, membranes, nucleotides, glycolysis, and others. Then, they were designed and synthesized in accordance with the groups. This reorganization involved all the genes at segment 2, which is difficult for traditional methods to accomplish. The reordered strains did not display significant growth defects, suggesting that gene order is not critical for life [22].

Despite the success in constructing a minimal genome, JCVI-syn3.0 had a significant growth defect and striking morphological variation. The doubling time of JCVI-syn3.0 (180 min) was observably longer than JCVI-syn1.0 (60 min), and cells appear to be polymorphic [28]. This suggests that JCVI-syn3.0 can survive and replicate with difficulty, but it lacks some important genes required for normal organisms. To identify genes required for normal cell division by repairing striking morphological variation in JCVI-syn3.0cells, the eight minimized genomic segments of JCVI-syn3.0 were individually replaced into the genome of JCVI-syn1.0. The segment that led to abnormal growth was identified and simplified again. Nineteen genes were added into JCVI-syn3.0 to generate JCVI-syn3A, which presented similar morphology compared with JCVI-syn1.0. Seven genes of the nineteen genes were necessarily together for normal cell division, including two known cell division genes, ftsZ and sepF, a hydrolase of unknown substrate, and four genes that encode membrane-associated proteins of unknown function [28].

With normal propagation and morphology, JCVI-syn3A provides a concise platform to study the basic structure of cells. Through the study of JCVI-syn3A, a comprehensive cell model can be constructed, including gene function, cell structure, dynamic regulation of life processes, etc. Zaida Luthey-Schulten and colleagues constructed a whole-cell fully kinetic model of JCVI-syn3A. Cryo-electron tomograms provided the cell geometry, including configuration of the circular chromosome and spatial distributions of ribosomes [29]. A near-complete metabolic network of JCVI-syn3A was assembled, in which 98% of enzymatic reactions were supported by annotation or experiment. The model generally reproduced the findings of experiments and found 30 essential genes with unknown functions [30]. After that, the function of all 452 protein-coding genes in JCVI-syn3A were annotated using evolutionary sequence analysis, protein structure prediction, interactomics, and genome architecture [31]. Additionally, they revealed how the cell balanced the demands of its metabolism, genetic information processes, and growth by the time-dependent behaviors of concentrations and reaction fluxes from stochastic–deterministic simulations over a cell cycle. They further analyzed the energy economy strategy of each metabolic process, including active transport of amino acids, nucleosides, and ions, and demonstrated how emergent imbalances cause transcription and translation rates to slow down [32].

Overall, the minimal genome provides an unprecedented platform for understanding the basic function of the genome. Obviously, the minimal genome is not a cluster of essential genes; it requires a deep understanding of gene function and gene interaction. In the minimization process of the *C. crescentus* genome, genetic features were reduced from 6290 to 799 [24,33,34]. A transposon insertions test showed that 81.5% of essential genes are equal to natural genes in functionality. However, they cannot support cell survival without the natural genome [24,35]. The functions of many elements have yet to be investigated. Despite the assistance of databases and software, the design, synthesis, and testing of minimal genomes remain challenging. The researchers further analyzed the energy economy strategy of each metabolic process, including active transport of amino acids, nucleosides, and ions. They also showed how emergent balances cause transcription and translation rates to slow down.

## 3. Genome Research Using Codon-Compressed Synthetic Genome

**Figure 3:**
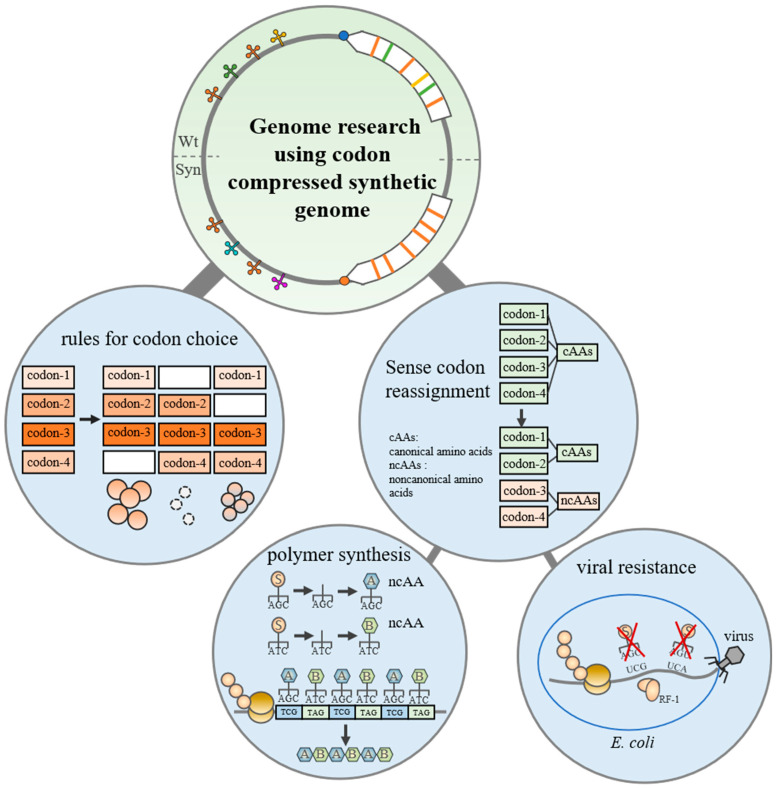
Genome research using a codon-compressed synthetic genome. Allowed and disallowed synonymous recoding schemes were tested and defined. The codon-compressed synthetic genome enables viral resistance and encoded polymer synthesis.

Due to genetic code degeneracy, synonymous codons can be swapped without affecting the amino acid sequence. It seems that there are some “redundant” codons that can be replaced by synonymous codons [36]. In most cases, it is feasible to alter partial codons of a single gene. However, at the genome scale, the replacement of synonymous codons is not always viable [37]. Synonymous codon alternatives can affect gene expression, and we have a poor understanding of the regulatory mechanisms [38]. Several theories have attempted to explain this effect, including that the replacement of synonymous codons disrupts regulatory elements and influences mRNA secondary structure [39,40,41,42]. The design of codon compression genomes provides a powerful approach for understanding the principle of codon choice (Figure 3). George M. Church et al. designed a 57-codon *E. coli* genome, in which 7 codons, including a stop codon (AGA, AGG, AGC, AGT, TTA, TTG, and TAG), were removed [9]. The genome was divided into 87 fragments, which were synthesized separately; 55 segments of them were tested individually and 91% of the tested essential genes were functioning. However, this genome was not fully assembled. Jason W. Chin and et al. created a variant of *E. coli* (Syn61) that contained a total synthetic 61-codon genome with 1 stop codon (TAG) and 2 serine codons (TCG and TCA) removed [8]. This synonymous codon compression scheme was picked from eight schemes which were tested and defined and allowed or disallowed beforehand [37]. The synthetic genome was assembled by iterative repetition of REXER, which is a technology coupling lambda-red recombination and positive/negative selection [43,44]. To reduce the difficulty of rewriting, both designs of codon compression genomes separated overlapping genes. Previous studies have found that overlap in genes is widespread and functionally integrated into prokaryotic, eukaryotic, and viral genomes [45,46,47]. Recoded target codons which fall into the overlapping region may disrupt regulatory elements, such as promoter and ribosome binding sites (RBS) [48,49]. The recoded genomes separated overlapping genes by duplicating the overlapping sequence and the 20-bp sequence upstream of the overlap, only recoding the ORF and maintaining the regulatory element. By the way, there is another modification that does not separate overlapping genes. It uses computational prediction to improve codons to maintain the functions of genes and regulatory elements [50]. So far, neither compression strategies guarantees gene expression directly because of knowledge limitations. Feasible synonymous recoding schemes need to be further investigated via experiments [37,51]. An increasing number of studies have concentrated on the annotation of regulatory elements and the function of RNA secondary structure, which can aid in the rational design of synonymous recodes [35,52]. Moreover, codon compression genomes refine the principle of codon choice and provide a flexible platform to study gene regulation and evolution.

Synthetic codon compression genomes in which codons enable reassignment expand biological functions by orthogonal translation systems (Figure 3) [53,54,55,56]. Non-canonical amino acids can be used to expand the structures and properties of peptides and proteins. However, protein synthesis is inefficient in vitro [57,58,59,60]. Previous work has shown that replaced and reassigned stop codon TAG in vivo can improve the production of target proteins which contain non-canonical amino acids [61,62]. Nevertheless, these processes will interact with the natural translation process, and more assignable codons are desired to introduce multiple non-canonical amino acids. Syn61∆3 was engineered from Syn61. It not only recoded the genome to free up TCG, TCA, or TAG codons in the open reading frames, but also deleted the tRNAs which decoded TCG, TCA, and the release factor 1, which, in turn, decoded TAG. Syn61∆3 provides an unmatched platform for the study of non-canonical amino acids. These codons can be reassigned to encode non-canonical amino acids (ncAA) and construct a mutually orthogonal aminoacyl-tRNA synthetase (aaRS)/tRNA pairs system. Researchers assigned TCG, TCA, and TAG codons to encode distinct ncAAs monomers. The corresponding non-canonical tetramers, hexamers, and octamers, as well as a non-natural macrocycle, were synthesized [63]. In addition, Syn61∆3 addressed a long-standing hypothesis that sense codon-compressed genomes are antiviral. Previous studies have shown that the *E. coli* strains which had all TAG stop codons replaced displayed increased resistance to the T7 bacteriophage [56]. Syn61∆3 exhibited complete resistance to a cocktail of viruses. It is due to this that mRNAs of viruses containing UCG, UCA, and UAG codons could not be completely translated in Syn61∆3; they are unreadable for the engineered translation system. The genome of Syn61∆3 showed the potential to study translation processes and improve industrial strains.

In conclusion, codon-compressed genomes provide an insight into studying codon usage, RNA structure, and the translation process. An environmental safety cell was created, the translation process of which was orthogonal to natural organisms and able to synthesize proteins containing three distinct non-canonical amino acids [64,65].

## 4. Genome Research Using Synthetic Yeast Genome

**Figure 4:**
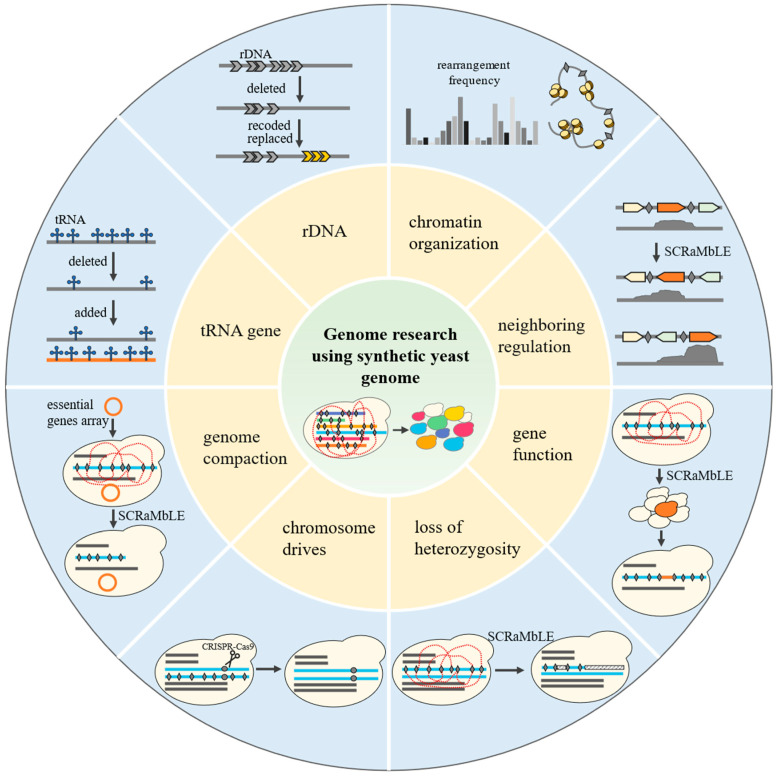
Genome research using a synthetic yeast genome. Synthetic yeast genome replaced tRNA and rDNA to research their function. Structure, function, and evolution of the genome were investigated by SCRaMbLE. Furthermore, the synthetic yeast genome was used to drive chromosomes and compact the genomes.

Sc2.0 is a project to synthesize the genome of *Saccharomyces cerevisiae*. Chromosomes of Sc2.0 were designed with BioStudio software which referenced the sequence of the S288C strain and were assembled by chemically synthesized fragments (Figure 4) [66]. So far, 9.5 single synthetic chromosomes have been constructed (synI, synII, synIII, synIV, synV, synVI, synVII, synIXR, synX and synXII) [10,11,12,13,14,15,16,17,18]. Strains harboring multiple synthetic chromosomes have also been constructed [67,68]. The design of Sc2.0 includes deletion of Ty elements, some introns, and subtelomeric repeats [69,70]; introduction of loxPsym sites; and replacement of telomeres [16], rDNA [18], and parts of codon sequences [16,71]. In addition, synthetic chromosomes have hundreds of loxPsym sites which can be recognized by Cre-EBD recombinase. With the induction of β-estradiol, the synthetic chromosome can generate a significant amount of large-scale structural variations (SCRaMbLE) [16,72].

Functional artificial chromosomes address some of the questions raised when they are designed: “Is it feasible to alter the copy number, location, and sequence of multi-copy elements, including subtelomeric repeat, tRNA, and rDNA?” Subtelomeric repeat sequences and yeast native telomeres were deleted in design, and universal sequences were placed on the new ends after deletion. This confirmed that it is feasible to change both the position and sequence of telomeres [62,73,74]. The deletion of subtelomeres shortens the distance between functional genes and telomeres. In most synthetic chromosomes (synII, synIII, synV, synIXR, synX and synXII), genes expressed normally. However, in synVI, core X elements of the universal telomere failed to buffer the telomere position effects fully, which led to an additional gene silencing, and the synVI strain displayed a growth defect [15]. In addition, ring synV, which deleted both chromosome telomeres, exhibited good fitness, while the frequency of spore viability was lower than in linear chromosomes [14]. All tRNAs on synthetic chromosomes were deleted. When strains contained one synthetic chromosome alone, tRNA genes could be deleted from the synthetic chromosome without growth defects. Meanwhile, when stains contained both synIII and synX, a combinatorial defect occurred and the strain showed an obvious growth defect at high temperatures. This was due to the insufficient expression of Swi3, which is a key positive inositol biosynthesis regulator located on synX. Swi3 contains two tandem UCG codons, and the deletion of tRNA CGA, which decoded the rare UCG serine codon on synIII, affected the translation of Swi3 [67]. Another study increased the copy number of tRNA genes; 275 additional nuclear tRNA genes were synthesized as a neo-chromosome in yeast. However, this seemed like a burden for cells; the host cells tended to increase ploidy or delete part of the tRNA neo-chromosome [75]. Moreover, rDNA was recoded and relocated in Sc2.0. During the construction of synXII, a rDNA seed fragment containing 1.2 or 2 copies of the rDNA unit and a hyg1 mutation were inserted at a natural or novel location. The rDNA copy number was coupled to the hyg1 copy number and expanded via hygromycin B selection. This implies that yeast can tolerate different copy numbers of rDNA, and rDNA copy numbers can increase spontaneously. rDNA can be removed from synXII to multicopy plasmids, the right arm of chromosome XV, or the right arm of chromosome III without growth rate defects [18]. However, the rDNA locus on the right arm of chromosome III imposed new constraints on the genome and affected the global 3D structure [76]. The transcribed spacer (ITS)region of rDNA, which is used as a DNA barcode to distinguish species, was replaced by the sequence from *Saccharomyces bayanus*, *Schizosaccharomyces pombe*, or *Candida albicans*. Only the ITS from *S. bayanus* supported cell survival and normal growth. These studies imply that yeast can tolerate changes in copy number, location, and sequence of multi-copy elements to a certain extent, but global rewriting requires further coordination and debugging with the relevant genes.

There is some structural engineering on a chromosome-wide scale occurring with synthetic chromosomes. According to the design principle of Sc2.0, synI was reduced by 21.6% compared with the wild type chrI. This 180 kbp chromosome is the smallest chromosome in the whole genome. Considering that a too-short length can negatively affect chromosome stability, synI was fused to another synthetic chromosome to ensure its stability [77]. To investigate whether the chrI performance would be affected by the alteration of the 3D environment, synI was fused to the shortest chromosome arm (chrIXR), the longest chromosome arm (chrIVR), and an intermediate-length chromosome arm (chrIIIR), respectively. None of the three chromosome fusion strategies affected strain growth. However, an unexpected loop was observed between the chrIR telomere and the HMR locus on chrIII, which was formed depending on silencing protein Sir3 [78]. For the largest synthetic yeast chromosome synIV, researchers flipped the linear orientation of chromosome arms. CEN4 was connected to the telomere-adjacent sequence, and UTC was added to the original CEN4 flanking sequence. This “Inside out” synIV revealed limited gene expression changes, showing only minor alterations in gene expression [13].

Besides the specific targeted engineering, synthetic chromosomes can generate random structural variations. SCRaMbLE led to inversions, deletions, duplication, translocation, and other complex structure variations at the designed sites. This makes it a flexible tool for studying genome structure and function. However, the rearrangement frequency of SCRaMbLE is not averaged at different positions, and the structure of the chromosome has a significant impact on the rearrangement frequency. By sequencing the SCRaMbLEd pool, the rearrangement pattern of the synthetic yeast chromosomes was revealed. The rearrangements tended to take place between loxPsym sites in chromatin-accessible and 3D spatially proximal regions [68]. Modifications to chromosome structure affect chromosome contact and rearrangement frequency. The circular chromosome enhanced inter-chromosomal contacts and increased recombination frequencies [79]. The deletion of the silent mating-type cassettes led to a loss of contacts on synIII, and the location of rDNA affected the global 3D structure of the genome [76]. In addition, Cre enzyme abundance and genome ploidy also influenced the rearrangement frequency [79].

The hundreds of loxPsym sites inserted on synthetic chromosomes allowed SCRaMbLE to generate a wide variety of structural variants with rich phenotypes. This provides a new method for studying genotypes and phenotypes. So far, two methods have been found to change gene expression: (1) gene deletion or duplication. Obviously, deletion or duplication of genes will directly affect gene expression and then affect phenotype. For example, a duplication of the gene YJR043 results in caffeine tolerance [80], a deletion of the gene YER161C results in alkali tolerance [81,82], deletions of the genes YBR219C and YBR220C result in hygromycin B resistance [83], and a deletion of the gene YLR131C results in ethanol tolerance [82]. Moreover, SCRaMbLE also serves as a tool to improve host strain or increase yield, and provides a platform for studying metabolic pathways [80,82,84,85,86,87,88,89,90]. (2) Neighborhood regulation: Differently from traditional studies, SCRaMbLE not only led to gene deletion and duplication, but also randomly changed the relative position of genes, which allowed us to use SCRaMbLE on synthetic chromosomes to study neighborhood regulation. The genomic neighborhood influences the expression of the genes within it [91]. However, it is difficult to systematically observe genes in different transcriptional contexts at the genome scale with other existing technologies; thus, SCRaMbLE provides a valuable platform. Luo et al. found that inversion of YLR131 increased ethanol tolerance [82]. Lars M. Steinmetz and colleagues analyzed 612 SCRaMbLEd genomic samples, and found that genomic rearrangements influence transcript isoform lengths and expression levels. These influences do not result from encoding in the CDS or 3 UTR sequence, but are caused by decoupled 3′UTR sequences and neighboring gene expressions which are perturbated by SCRaMbLEing. Researchers identified these regulatory features so that the alterations could be predicted and transcript length could be controlled by the positional context in genome engineering [92].

SCRaMbLE can randomly generate large-scale genomic structural variation. This allows us to study the gene structural changes and associated phenotypic evolution. *S. cerevisiae* containing a synthetic chromosome can mate with the Y12 sake-brewing strain and *S. paradoxus* [80,93]. Using interspecies SCRaMbLEing, a wide variety of phenotypes was rapidly provided for heterozygous diploid yeast strains and aneuploidy, and different levels of loss of heterozygosity (LOH) were observed, including short-range LOH in part of the synthetic chromosome, long-range LOH in an arm of the synthetic chromosome, and whole-chromosome LOH. By the way, some LOHs can lead to rapamycin resistance in synthetic yeast [94]. Another type of whole-chromosome LOH is not induced by SCRaMbLE, but is produced by a chromosome drive. In a diploid strain containing synX and chrX, synX can be eliminated efficiently by generating a double-strand break around the centromere of synX with CRISPR-Cas9. Interestingly, most of these 2n − 1 strains can spontaneously recover to 2n strains, though by duplicating the counterpart homologous chromosome chrX during sexual reproduction. A chromosome drive with non-Mendelian-biased inheritance on a chromosomal scale was confirmed in synthetic strains [95].

Another interesting method for engineering synthetic genomes is to use SCRaMbLE to compact genomes in order to explore the basic function of genomes. It also provides a possible method for top-down construction of minimal genomes. To verify the feasibility of this method, a strain containing synthetic chromosome III and a centromeric plasmid which clustered all essential genes of chromosome III were used to be reduced to compact a synthetic genome. With the essential gene plasmid, 35.7% of the length of synthetic chromosome III was deleted by SCRaMbLEing [96]. Luo, Z., et al. compacted the left arm of synthetic chromosome XII. With an essential gene array, the strain deleted 39 out of the total 65 nonessential genes in chromosome XIIL by SCRaMbLEing. This genome-compacted strain did not exhibit growth defects in a rich medium. Using this method, several genes needing underspecified conditions were also identified [97]. In addition, this strategy revealed synthetic lethal interaction regions in the genome. Although the essential gene array greatly improves the compacting ability of the synthetic chromosome, it seems that regions on the synthetic chromosome cannot be deleted arbitrarily with the addition of essential genes. In synthetic chromosome III, a region which only harbors six nonessential genes cannot be deleted by SCRaMbLEing. This region corresponds to a hidden synthetic lethal interaction. Researchers also designed three essential gene plasmids with different gene orders: the genomic-direction plasmid, the same-direction plasmid, and the random-direction plasmid. This gene order reorganization had little effect on gene expression [96]. Those studies established a framework to compact genomes via SCRaMbLEing.

In general, although the rewriting of the SC2.0 genome was relatively conservative, it still raised and addressed some key questions by rewriting tRNA and rDNA. Specially, the loxPsym sites introduced upon design confer the ability of the synthetic genome, which can generate extensive rearrangements. Moreover, a pan-genome neo-chromosome was created that expanded the genome’s content in order to study genetic diversity. The chromosomes are compatible with the design principle of Sc2.0, which can generate a wide variety of phenotypes by SCRaMbLEing [98]. SCRaMbLE can generate massive rearrangements at a genome-wide scale, which makes the synthetic yeast genome an excellent platform to study genomic structural variation.

## 5. Conclusions

Large-scale genome rewriting offers a new perspective for studying genome biology. Most of the synthetic genomes were designed to create a more controlled and easier-to-use genome engineering platform by systematic modifications of natural genomes. Through cycles of design–build–test of synthetic genomes and the following engineering of synthetic genomes, many fundamental questions of genome biology can be investigated. Meanwhile, some novel biological functions that cannot occur in nature can be achieved by designer genomes. Although the field of synthetic genomics has just begun to develop, its broad prospects can be predicted.

## Data Availability

Not applicable.
